# Fatal Case of Epistaxis

**Published:** 1831-02

**Authors:** F. Sievwright


### EPISTAXIS.

*Fatal Case of Epistaxis.* By F. Sievwright, Esq.*

Corporal James Ambler, aged forty; depôt staff corporal, H. M. 13th Light Infantry; was admitted into hospital, at seven A.M., 28th September, 1826, with profuse bleeding from the nose, confined almost entirely to the right nostril. It commenced two hours previous, in his barrack room; and it appears he lost a large quantity of blood before he was brought to the hospital. Upon inquiry, he had been in a state of inebriety for several days, and, on the 26th September, had fallen down a flight of stone steps in that condition, and injured the head and nose; marks of violence being evident on both. No hemorrhage, however, took place until this morning (28th September.) He was a stout-made man, with a remarkably short neck.

Seven A.M. Wet cloths were applied to the head, and an attempt made to plug up the nostrils. A sense of suffocation, however, followed, and they were withdrawn. A dossil, dipped in Spt. rectificati, was then introduced up the right nostril, which, for a short time, succeeded in checking the hemorrhage. Afterwards a strong solution of Sulphat. Cupri. was repeatedly thrown up the nostrils by means of a syringe; part of which being inadvertently swallowed, produced great sickness at stomach. A probe, and ligature attached, was unsuccessfully attempted to be passed through the nostril into the mouth.

Nine A.M. When the bleeding ceased, a small quantity of wine and water was given; the patient was exposed freely to a current of cool air, a damp cold sheet wrapped about the head and shoulders, and a cool acidulated drink allowed him.

Five P.M. Since last report, the hemorrhage returned for a short time, but has again ceased of its own accord. Pulse very languid, surface warm; has had some sleep; bowels not moved since admission. A purgative enema ordered; and during the day he took repeated doses of the Dec. Cinchonæ cum Acid. Sulph. dilut. The blood from the commencement

* Trans. Med. and Phys. Society, Calcutta.

had evidently been arterial, at times coming by drops in quick succession, at others pouring forth in one continued stream.

29th Sept. Passed a quiet night. No return of hemorrhage. Bowels still confined, which was remedied by the exhibition of a purgative. The decoction of bark, &c. continued, and he took some arrowroot and wine.

Ten A.M. Hemorrhage afresh, and to a considerable extent. Had a draught with ʒi. Spt. Ætheris Sulph.; some arrowroot and port wine. Bottles of hot water were applied to the hands and feet, and the draught was repeated as follows: ℞. Spt. Ammoniæ Aromat. ʒi.; Spt. Lavandulæ comp. Spt. Ætheris Sulph. *aa* gtt. xi.; Aquæ ℥i. Fiat haustus statim sumendus.

One P.M. The bleeding has stopped since eleven o'clock. Pulse very weak; feels occasionally very cold, accompanied by shivering. Bath with acid continued; in addition to which he is ordered ʒi. Mist. Camphoræ every hour.

Five P.M. Pulse fuller, more animated. The sense of cold attended by shivering has disappeared; feels greatly better, and seems anxious to sleep. An anodyne draught ordered at eight o'clock. Every thing continued quiet, and he dropped asleep.

30th Sept. Passed a quiet night. At thirty-five minutes past three o'clock this morning, experienced a sudden return of hemorrhage, and almost immediately expired.

*Dissection.* Seven hours after death, on removing the skullcap, it was remarked how very easily this was accomplished after the bones had been sawed through, the dura mater adhering to them in an unusually slight degree: this proved to be the case generally, even to the base of the cranium; so much so, that, with no other assistance than what the fingers afforded, the whole of the dura mater was removed with as much facility as if the head had been macerated in water for six weeks.

At the first glance of the brain, a very different appearance presented itself from what might have been expected, (a very deceitful appearance,) that of congestion! On examining the parts narrowly, however, a great many of the blood-vessels were quite empty; others contained a colourless fluid. There was a quantity of serous fluid on the surface of the brain, and a large proportion in the different cavities, in the lateral ventricles particularly. A quantity of liquid blood, intermixed with serous fluid, was effused on the left occiput, between the cranium and the membranes; and at the base of the skull there were at least two ounces of limpid water. No marks of fracture were here to be seen.

The skin and muscles were then dissected back from the face, and, by means of a saw, a section made of the head. A large coagulum of blood was traced from the left nostril, communicating with the frontal sinus: there was a smaller coagulum on the opposite side, which, in a similar manner, appeared to communicate with the frontal sinus. Extensive marks of severe contusion appeared on and in the vicinity of the ossa nasi. Prosecuting the inquiry, the antrum maxillare of the right side was examined, by cutting through and destroying the orbitary plate. The entire cavity was filled by a large coagulum of a dark brown colour, and which had evidently been formed there for some time; at the date of, or soon after, the accident. This opinion is founded upon its colour, and from the circumstance of a quantity of coagulable lymph being seen on its surface. The antrum on the left side was also looked at, by breaking down the orbitary process, and a smaller coagulum of the same description was found therein. The substance of the brain was firm. The quantity of serous fluid effused exceeded six ounces.

